# The use of soft contact lens for intraocular foreign body removal to avoid inadvertent cataract formation

**DOI:** 10.1093/jscr/rjab404

**Published:** 2021-10-05

**Authors:** Maroun Eid, Reeda Bou Said, Elias Jarade

**Affiliations:** Ophthalmology Department, Lebanese University, Beirut, Lebanon; Ophthalmology Department, Lebanese University, Beirut, Lebanon; Ophthalmology Department, Lebanese University, Beirut, Lebanon; Corea and Refractive Surgery, Ophthalmology Department, Lebanese University, Beirut, Lebanon

## Abstract

Ocular injuries carry a significant morbidity worldwide. Visual outcomes vary depending on the mechanism of injury, the damage on arrival and the surgical technique used. The presence of an intraocular foreign body further complicates matters, due to its constituents, the infectious potential, and/or damage to intraocular structures. Another described problem is the inadvertent cataract formation due to contact with the natural lens during removal of the foreign body. The purpose of this paper is to describe a case with a mechanical foreign body lodging in the posterior chamber without harming the lens. The surgical challenge was to remove it without causing cataractous changes. Therefore, a soft contact lens was used to form a protective layer between the foreign body and the natural lens of the eye allowing successful removal of the foreign body.

## INTRODUCTION

Ocular trauma is an important cause of morbidity. About 55 million ocular injuries occur annually [1], with 8–25% of open globe injuries associated with intraocular foreign body (IOFB; [2]). IOFBs vary in nature and location within the eye. Metallic IOFB are the most common and require removal to avoid complications related to their constituents [2]. Classically, plain X-rays were the only available tool used to detect IOFB, but B-scan ultrasonography (B scan) and computed tomography (CT) scans are now the standard methods for diagnosis, as well as for determining possible associated complications including retinal detachment [3].

‘First, do no harm’ is a doctrine as old as medicine itself. It addresses iatrogenic problems, complications inadvertently created by well-intentioned medical or surgical therapies [4]. Among the most common causes of iatrogenic cataract are medications, radiation and prior intraocular surgeries [5]. Procedures most commonly associated with iatrogenic cataracts in the literature include vitrectomies, trabeculectomies, penetrating keratoplasties and peripheral iridectomies [5].

One that is frequently overlooked is the surgical procedure performed to remove a crystalline-lens-sparing anterior-chamber foreign body. As a matter of fact, the presence of anterior-chamber IOFB without cataract formation should alert the surgeon to the importance of its removal without injuring the anterior capsule, and undeniably the corneal endothelium. An ophthalmic viscosurgical device has been classically used to help minimize trauma while manipulating the foreign body, the typical approach is with an adjacent paracentesis. The instruments utilized vary depending on the size of the IOFB including Rappazzo forceps and straight forceps. Aspiration can be attempted with very small IOFBs [2].

The surgical technique described below addresses the aforementioned problem, allowing safe manipulation inside the anterior chamber thus decreasing the risk of unintentional contact with the anterior surface of the crystalline-lens.

## SURGICAL TECHNIQUE DESCRIPTION

**Figure 1:**
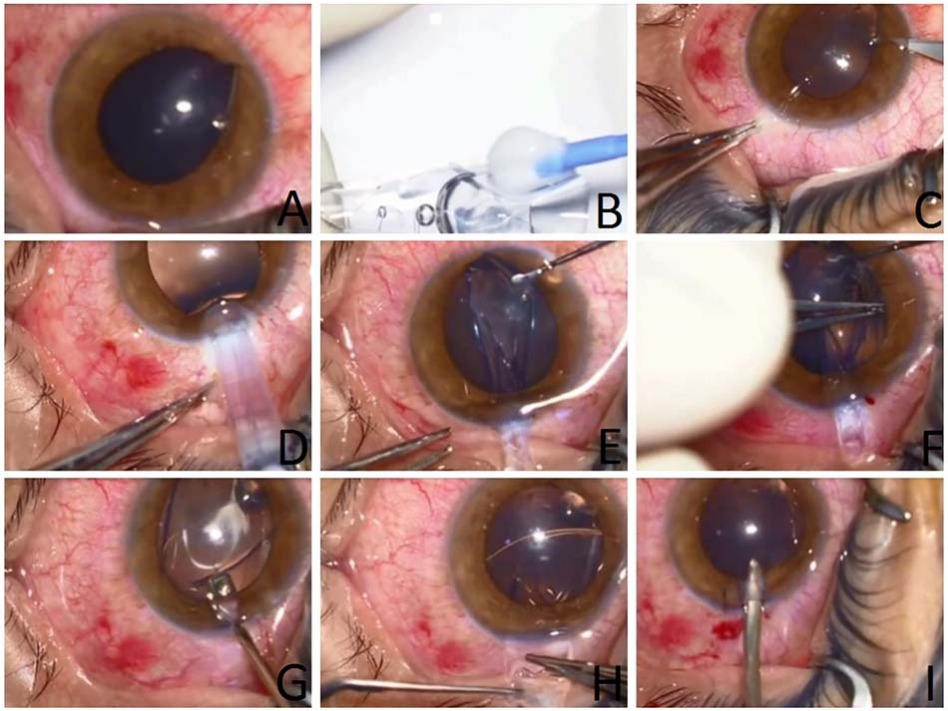
Images from the intraoperative use of soft contact lens to aid in foreign body removal. (**A**) Initial appearance showing location of foreign body passing through the inferior cornea and lodging behind the iris. (**B**) Preparing the soft contact lens with the Push-type Visian ICL STAAR injector. (**C**) Paracentesis next to the foreign body entry location. (**D**) Injection of the lens through the superior main corneal incision. (**E**) Adjusting the position of the SCL using a spatula. (**F**) Removal of the foreign body through its corneal entry site using McPherson Tying Forceps. (**G** and **H**) Careful removal of the soft contact lens. (**I**) Removal of Viscoelastic from the AC using a Simcoe I/A cannula.

This is the case of a long metallic foreign body penetrating the anterior chamber of the left eye through a corneal wound (at 9 o’clock) and lodging behind the iris (between 7 and 8 o’clock) with an apparently intact crystalline-lens (Fig. 1). The position of the foreign body between the iris and the anterior lens capsule makes the risk of damaging the lens critically high while removing it.

A small side incision was done—using 15 degree super sharp blade—adjacent to the position where the foreign body was at close proximity to the lens (7 o’clock). A 1.4% sodium hyaluronate viscoelastic solution was carefully injected into the anterior chamber. In order to protect the natural lens during foreign body extraction, a soft contact lens was injected into the anterior chamber using a Push-type Visian implantable collamer lens STAAR® (ICL STAAR) injector through a 3.2 mm superior corneal incision. A spatula was introduced through the side incision to adjust the position of the soft contact lens (SCL) at the interface between the foreign body and the anterior lens capsule. Then using an angled McPherson Tying Forceps the foreign body was removed through its corneal entry site.

The SCL is fragile and tears easily; so it was pushed inside the eye with a spatula and then the main incision was enlarged to facilitate manipulation and hence smooth removal of the contact lens without tearing it. Viscoelastic solution was injected underneath followed by a bimanual stepwise removal of the SCL using 2 angled McPherson Forceps. Finally, Viscoelastic was removed from the anterior chamber (AC) using a Simcoe I/A cannula and the superior main corneal incision closed with 2 interrupted 10–0 nylon sutures.

## DISCUSSION

Crystalline-lens injury and subsequently induced cataract is among the most common complications of surgical removal of intracameral foreign bodies. We are not aware of another major work proposing a way to prevent this relatively frequent complication.

In the event of an intracameral foreign body, several factors can make the mission of removing it more difficult. In most cases, a significant corneal wound may be present with associated corneal infiltration, edema and haze, making the surgical view more difficult and rendering careful surgical manipulation and avoidance of contact with the natural lens more challenging. Therefore, such cases increase the need for an intervening smooth surface allowing safer removal of the foreign body in these suboptimal operative conditions. Moreover, in certain circumstances related to the shape and location of the foreign body, things can be more critical. For example, in the case of a filiform metallic foreign body or if a part of the foreign body is lodged under the iris, the extent of penetration or extension is very difficult to appreciate, and very subtle movements can lead to loss of capsular integrity and cataract formation.

In this paper, we described a new technique to overcome these problems that consists of the use of SCL inserted into the AC forming a soft barrier to protect the natural crystalline-lens and allowing a safe removal of the long metallic FB without touching the anterior capsule of the natural lens. Postoperatively, the patient recovered 20/20 vision in that eye with long-term (>2 years) follow up. The crystalline-lens remained clear throughout this time with no signs of anterior-chamber infection or inflammation.

The use of Implantable Collamer® lens implants (ICL) as alternative to SCL in these cases is also to be considered, given that these implants are made of collagen-based material; they are soft and pliable, and more stable than SCL, making their manipulation in the eye easier. When compared with SCL, the latter are kept in solutions that are toxic to the corneal endothelium and thus require thorough washing with balanced salt solution. However, ICL are thicker which may render their placement more complicated in particular scenarios. Moreover, they are more expensive and less available than SCL. Soft contact lenses are cheap and readily available which may render their use more practical.

In conclusion, we report a novel surgical technique to prevent iatrogenic cataract formation, a way to protect the anterior lens capsule and prevent contact with a foreign body while removing it.

## CONFLICT OF INTEREST STATEMENT

None declared.
